# Letter from the Editor in Chief

**DOI:** 10.19102/icrm.2021.120608

**Published:** 2021-06-15

**Authors:** Moussa Mansour


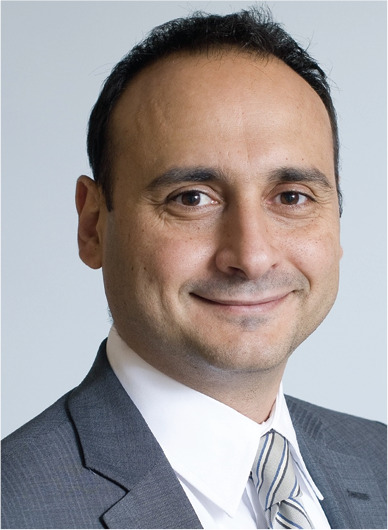


Dear readers,

At the recent annual scientific meeting of the American College of Cardiology, several late-breaking clinical trials were presented, including the Left Atrial Appendage Occlusion Study (LAAOS III), which evaluated the efficacy and safety of left atrial appendage (LAA) exclusion in patients with atrial fibrillation (AF) and CHA_2_DS_2_-VASc scores of at least two points undergoing cardiac surgery for another reason. More than 4,750 patients were randomized to either surgical exclusion of the LAA plus continuation of oral anticoagulation or to oral anticoagulation alone. The primary endpoint was the occurrence of ischemic stroke or systemic embolism, documented in 4.8% of the LAA occlusion group and 7.0% of the no-occlusion group (hazard ratio: 0.67, 95% confidence interval: 0.53–0.85; p = 0.001). Exclusion of the LAA was not associated with an increased risk during the cardiac surgery.

LAAOS III is a landmark study^1^ and will change clinical practice; from here on, patients with AF undergoing cardiac surgery should have the LAA excluded at the time of the surgery. Some study limitations exist, however, including the lack of a standardized technique for LAA exclusion. At least five different techniques were used to occlude the appendage, and no imaging studies were performed after surgery to assess the success of LAA closure. Knowing that some surgical techniques can lead to retention of an LAA stump,^2^ it is important to standardize LAA occlusion and confirm its success. Another limitation is that AF ablation was performed in about one-third of study participants and the effect of this fact on the results is unclear.

One important point not addressed by LAAOS III is the effect of surgical LAA exclusion on stroke prevention without the use of oral anticoagulation. While data showing the benefit of oral anticoagulants are robust, prior real-world experience with these medications has revealed that noncompliance and bleeding rates are not trivial.^3^ In LAAOS III as well, the rate of major bleeding was around 10%. As a result, it is important to quantify the magnitude of the incremental effect of oral anticoagulation on top of LAA exclusion, if there is any, on stroke prevention.

Best wishes for a healthy and relaxing summer.

Sincerely,


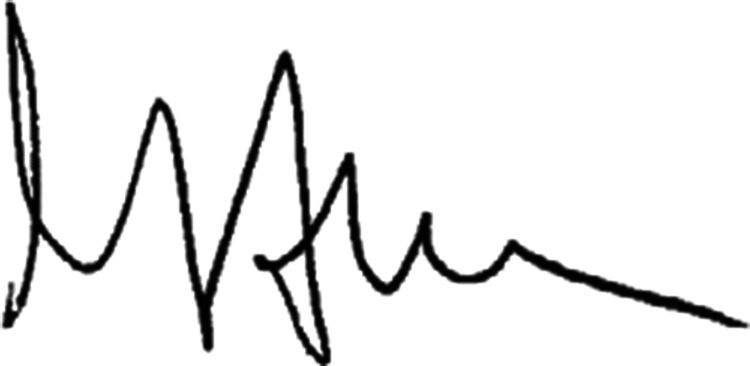


Moussa Mansour, md, fhrs, facc

Editor in Chief

*The Journal of Innovations in Cardiac Rhythm Management*

MMansour@InnovationsInCRM.com

Director, Atrial Fibrillation Program

Jeremy Ruskin and Dan Starks Endowed Chair in Cardiology

Massachusetts General Hospital

Boston, MA 02114
